# Risk factors analysis and risk prediction model establishment for heterotopic ossification after open reduction and internal fixation of distal humeral fractures: a retrospective cohort study

**DOI:** 10.3389/fmed.2026.1810326

**Published:** 2026-07-29

**Authors:** Qiming Liu, YuHai Wang, Zilin Zhang, Teng Ma, Weiwei Guo, Yue Qin, Hao Zhang, Min Zhang

**Affiliations:** 1 Department of Trauma and Orthopedics, General Hospital of Ningxia Medical University, Yinchuan, Ningxia, China; 2 Department of Trauma and Orthopedics, People’s Hospital of Ningxia Hui Autonomous Region, Yinchuan, Ningxia, China; 3 Department of Trauma and Orthopedics, Yinchuan First People’s Hospital, Yinchuan, Ningxia, China; 4 Department of Cardiology, General Hospital of Ningxia Medical University, Yinchuan, Ningxia, China

**Keywords:** distal, heterotopic, humeral fractures, nomograms, ossification, risk factors

## Abstract

**Objective:**

To identify independent risk factors for heterotopic ossification (HO) after open reduction and internal fixation (ORIF) of distal humeral fractures, and to construct and validate a clinically applicable risk prediction model.

**Methods:**

This retrospective cohort study included 826 patients with distal humeral fractures who underwent ORIF in 3 Grade A tertiary hospitals. Multi-dimensional baseline data were collected. The primary outcome was the occurrence of HO within 6 months after surgery. Potential risk factors were screened by univariate analysis and multivariate Logistic regression. The model was internally validated using Bootstrap resampling (1,000 iterations) to estimate the mean area under the receiver operating characteristic curve (AUC), and temporally validated using a cohort of 186 patients enrolled from the same tertiary hospital (temporal external validation). Receiver operating characteristic (ROC) curve, calibration curve and decision curve analysis (DCA) were used to evaluate the model’s performance. A nomogram was also constructed for visualization.

**Results:**

The overall incidence of postoperative HO was 23.7%. Age ≥ 60 years, high-energy injury, comminuted fracture, extensive intraoperative soft tissue dissection, postoperative immobilization ≥ 4 weeks and postoperative infection were independent risk factors (all *P* < 0.001). The internal validation AUC of the model was 0.846, and the external validation AUC was 0.823. The calibration was good (Hosmer-Lemeshow test, *P* = 0.610). When the risk threshold was between 8 and 75%, the clinical net benefit of the model was superior to traditional strategies. The nomogram could intuitively predict the risk of HO.

**Conclusion:**

This study identified the core risk factors for HO. The established prediction model can serve as a preliminary risk-stratification tool for HO. Further prospective studies and geographically distinct external validation are still needed before this model can be applied in routine clinical practice.

## 1 Introduction

Distal humeral fractures account for 2–6% of all fractures. They are one of the most common intra-articular fractures of the upper extremity. These fractures are often caused by high-energy injuries (such as traffic accidents and falls from heights) or low-energy injuries. The core goal of treatment is to restore the anatomical structure and mechanical stability of the elbow joint, so as to maximize the preservation of joint function (1–3). Open reduction and internal fixation (ORIF) has become the standard treatment for distal humeral fractures. It can accurately reduce the articular surface and firmly fix the fracture end. This significantly reduces the incidence of complications such as nonunion and malunion (4, 5). However, heterotopic ossification (HO) is a common complication after ORIF. It seriously affects the prognosis of patients. The reported incidence of HO varies greatly in previous studies, ranging from 15 to 40%(6–8). HO refers to the formation of mature bone tissue in soft tissues outside the normal bone tissue. It can cause elbow joint pain and limited range of motion. In severe cases, it may even lead to joint ankylosis, which significantly reduces the quality of life of patients and increases the risk of secondary surgery and medical burden (9, 10).

At present, the pathogenesis of HO has not been fully clarified. Existing studies suggest that it may be related to the excessive activation of local inflammatory response, abnormal recruitment and differentiation of osteoblasts, and dysregulation of cytokines such as bone morphogenetic proteins (BMPs) (11–13). Although scholars at home and abroad have carried out a series of studies on the risk factors of HO after ORIF of distal humeral fractures, there are obvious differences in the research conclusions. For example, some studies believe that age is a risk factor for HO (14, 15), while others have not found an association between age and HO (16, 17). Regarding the impact of surgical timing on HO, some studies suggest that early surgery can reduce the risk of HO (18), but others believe that surgical timing has no significant correlation with the incidence of HO (19). This inconsistency in research results is mainly due to factors such as small sample size, heterogeneity in study design, and incomplete inclusion of risk factors. As a result, the core independent risk factors for HO have not been clarified.

More importantly, existing prediction models for HO after elbow or distal humerus fracture surgery suffer from methodological limitations that restrict their clinical utility. First, most prior models were derived from small, single-center cohorts without external validation, leading to poor generalizability and potential overfitting (20). Second, variable selection was often incomplete or arbitrary, lacking rigorous univariate and multivariate screening to identify truly independent predictors. Third, few studies performed comprehensive performance assessment including discrimination, calibration, and decision curve analysis; thus, the clinical net benefit of these models remains unproven. Finally, nearly all models were generic for HO after any fracture, rather than specifically designed for patients undergoing ORIF for distal humeral fractures, resulting in low specificity and limited applicability in routine clinical practice.

To address these well-documented flaws, the present study was designed with rigorous methodology: we used a large multicenter retrospective cohort, performed strict variable selection via univariate and multivariate logistic regression, implemented both internal Bootstrap validation and independent external validation, comprehensively evaluated model performance using ROC, calibration curves, and DCA, and developed a disease-specific nomogram for HO after distal humeral fracture ORIF. By directly overcoming the methodological weaknesses of prior models, this study provides a more reliable, generalizable, and clinically practical tool for individualized risk assessment.

## 2 Materials and methods

### 2.1 Study design and participants

This was a multicenter retrospective cohort study. The research subjects were patients with distal humeral fractures who underwent open reduction and internal fixation (ORIF) in the orthopedics departments of three tertiary grade A hospitals (General Hospital of Ningxia Medical University, People’s Hospital of Ningxia Hui Autonomous Region, and Yinchuan First People’s Hospital, Ningxia) from January 2018 to December 2023. A consecutive sampling method was utilized, meaning every patient meeting the inclusion criteria during this timeframe was considered for inclusion, rather than selecting specific cases. All participating medical institutions have rich experience in orthopedic diagnosis and treatment, and the diagnosis and treatment processes are all standardized. This study was approved by the Ethics Committee of the General Hospital of Ningxia Medical University. This single ethics approval centrally covered and coordinated the ethical review process for all three participating tertiary hospitals to ensure uniform ethical standards across all study sites.

#### 2.1.1 Inclusion criteria

➀ Diagnosis of distal humeral fracture (AO/OTA classification: 13-A, 13-B, 13-C type) confirmed by X-ray and CT examination; ➁ Age ≥ 18 years old; ➂ Undergoing ORIF, with surgical approaches including olecranon osteotomy approach or lateral approach; ➃ Complete clinical data (including demographic characteristics, fracture-related information, surgical records, postoperative follow-up data, etc.); ➄ Postoperative follow-up time ≥ 6 months (based on the research evidence that the peak incidence of HO is mostly 3–6 months after surgery, to ensure that HO can be clearly diagnosed) (21, 22).

#### 2.1.2 Exclusion criteria

➀ Complicated with fractures of other parts (such as ulna and radius fractures) or severe organ injuries (such as craniocerebral, thoracic and abdominal organ injuries); ➁ Preoperative presence of heterotopic ossification, osteoporosis, bone tumors, metabolic bone diseases and other diseases affecting bone metabolism; ➂ Postoperative occurrence of severe complications (such as internal fixation failure, vascular and nerve injuries requiring reoperation); ➃ Incomplete follow-up data or loss to follow-up (follow-up rate < 80%); ➄ Complicated with mental illness, cognitive impairment or severe underlying diseases (such as advanced tumors, severe cardio-cerebrovascular diseases) that cannot cooperate with follow-up and functional evaluation; ➅ Pregnant or lactating women.

#### 2.1.3 Sample size estimation

PASS 15.0 software was used for sample size estimation. Referring to previous studies on heterotopic ossification after distal humeral fractures (7), the anticipated incidence of HO (event rate) was set at 20%. Given that the primary objective was to identify independent risk factors via multivariable logistic regression, we specified an odds ratio (OR) of 2.0, which was conservatively estimated based on the effect size of key binary risk factors identified in prior literature, such as high-energy injury versus low-energy injury (18).

To ensure adequate precision for the multivariable model, which initially included 8 candidate predictors (corresponding to approximately 10 degrees of freedom), we adopted an Events Per Variable (EPV) approach. With an expected HO incidence of 20% and aiming for a minimum of 10–15 events per variable, the required sample size was calculated. Under the assumptions of a two-sided α = 0.05 and a statistical power (1-β) of 90%, the estimated minimum sample size was 748 cases. Considering a 10% attrition rate for loss to follow-up, the final planned enrollment was 826 cases, which exceeded the minimum requirement and ensured sufficient statistical power for the subsequent multivariate analysis. The validation cohort comprised 186 patients who underwent ORIF between January 2019 and June 2023, enrolled from General Hospital of Ningxia Medical University. Although the overall study period overlaps with the main cohort (2018–2023), the two cohorts were strictly separated in data collection and patient enrollment, with no overlapping individuals and no shared surgical or rehabilitation periods. This design adopts temporal external validation, which helps reduce selection bias and improve generalizability. Notably, this cohort does not constitute full independent geographic external validation.

### 2.2 Outcome definition

The primary outcome was the occurrence of postoperative heterotopic ossification (HO). The diagnosis of HO adopted the standard combining clinical symptoms and imaging examinations, and both criteria must be met simultaneously: (23): ➀ Clinical symptoms: Elbow joint pain and limited movement, excluding other causes such as infection and internal fixation loosening; ➁ Imaging examinations: At 6 months after surgery, X-ray or CT examination showed new bone tissue in the soft tissues around the distal humerus, and there was no continuity with the normal bone tissue. Two orthopedic doctors with more than 5 years of clinical experience independently diagnosed HO. Crucially, these assessors were blinded to the patients’ baseline risk factors, surgical details, and group assignments to minimize potential diagnostic bias. In case of disputes, a third orthopedic expert made the final judgment.

It is important to note that the primary outcome of this study was defined as a binary variable (presence or absence of HO) based on radiographic evidence. We did not employ a specific elbow classification system (e.g., Hastings and Graham classification) to grade the severity or anatomical location of HO. This decision was made because the primary aim of this study was to construct a risk prediction model for the incidence of HO, rather than for its clinical severity. Furthermore, given that all included patients were required to have a minimum follow-up of 6 months, we prioritized the detection of HO formation over its morphological characterization.

### 2.3 Collection of baseline data

Through a standardized data extraction form, researchers who received unified training extracted patients’ baseline data from the electronic medical record system. The specific data included the following four categories:

#### 2.3.1 Demographic characteristics

Age, gender, body mass index (BMI), smoking history (smoking ≥ 1 cigarette/day for ≥ 1 year was considered as having a smoking history), drinking history (drinking ≥ 1 time/week for ≥ 1 year was considered as having a drinking history), and comorbidities (such as diabetes, hypertension, rheumatoid arthritis, etc., confirmed by diagnosis in medical records).

#### 2.3.2 Fracture-related indicators

Cause of fracture (high-energy injury: traffic accident, fall from height, heavy object impact; low-energy injury: fall, minor trauma), fracture AO/OTA classification (AO/OTA classification was determined by two senior orthopedic surgeons based on preoperative CT scans, with disagreements resolved by consensus), whether it was a comminuted fracture (≥3 fracture fragments were considered as comminuted fractures), whether complicated with elbow dislocation, time from fracture to surgery (time interval from fracture occurrence to the start of surgery).

#### 2.3.3 Surgery-related indicators

Surgical approach (olecranon osteotomy approach, lateral approach), operation time (time from skin incision to suture completion), intraoperative blood loss, range of soft tissue dissection (Graded intraoperatively by the operating surgeon as extensive and limited. Extensive: dissection range exceeding 5 cm from the fracture end or involving important ligament insertions around the elbow joint; limited: dissection range ≤ 5 cm from the fracture end without involving important ligament insertions, determined by the surgeon according to the surgical record), type of internal fixation (plate and screw, intramedullary nail), whether bone grafting was performed (autologous bone grafting or allogeneic bone grafting), professional title of the surgeon (chief physician/associate chief physician, attending physician, resident physician).

#### 2.3.4 Postoperative management indicators

Whether to use drugs for preventing HO after surgery: Non-steroidal anti-inflammatory drugs (NSAIDs) were administered for prophylaxis. Specifically, this was defined as the prescription and documented intake of either celecoxib (200 mg daily) or ibuprofen (400 mg three times daily) for ≥ 3 consecutive days postoperatively. Information regarding the specific drug type, dosage, and administration schedule was retrieved from electronic medication records and nursing charts. Patient compliance was verified through daily nursing documentation and outpatient follow-up records, ensuring that doses were not missed for more than one consecutive day; otherwise, the patient was classified as non-compliant and excluded from the “NSAID use” category. Intermittent or PRN (as-needed) use was explicitly excluded from the “NSAID use” definition.

Other indicators included postoperative immobilization time (Extracted from rehabilitation records as the number of weeks the elbow was immobilized postoperatively), start time of rehabilitation training (time of first standardized rehabilitation training after surgery), whether postoperative infection occurred (Diagnosed according to CDC criteria, including purulent drainage, positive cultures, or localized signs of infection with systemic response), postoperative drainage (whether to place a drainage tube, indwelling time of the drainage tube).

### 2.4 Data collection and quality control

#### 2.4.1 Data collection method

A double-check system was adopted. After one researcher extracted the data, another researcher reviewed the data to ensure the accuracy of data extraction. Before analysis, missingness was examined for all variables. Variables with a missing rate ≥ 5% were excluded from the final analysis; these included preoperative elbow range of motion (6.2% missing) and preoperative grip strength (5.7% missing). For variables with missingness < 5%, multiple imputation by chained equations (MICE) was performed in SPSS 26.0. The variables subjected to imputation were smoking history (2.8% missing), intraoperative blood loss (2.1% missing), and postoperative rehabilitation start time (1.5% missing). The imputation model included all candidate predictors entered into the multivariate logistic regression as well as the outcome variable (HO occurrence). Five imputed datasets were generated, and pooled estimates were used for final regression analyses. No significant differences in key baseline characteristics were observed between complete and imputed data (all *P* > 0.05).

#### 2.4.2 Quality control measures

➀ Before the start of the study, all data extractors were uniformly trained to explain the standards and methods of data extraction to ensure the standardization of the extraction process; ➁ For key indicators such as HO diagnosis, a double independent judgment + third-party arbitration method was adopted to reduce diagnostic bias; ➂ Regular spot checks were conducted on the extracted data to check the completeness and accuracy of the data, and problems were corrected in a timely manner; ➃ EpiData 3.1 software was used to establish a database, and logical verification functions were set during data entry to avoid entry errors.

### 2.5 Statistical methods

SPSS 26.0, R 4.2.0 and MedCalc 19.0 software were used for statistical analysis. The test level α = 0.05 (two-sided).

Normality test (Shapiro-Wilk test) was first performed for continuous data. Continuous data conforming to normal distribution were described as mean ± standard deviation (x ± s), and independent sample *t*-test was used for inter-group comparison; continuous data not conforming to normal distribution were described as median (interquartile range) [M (Q1, Q3)], and Mann-Whitney U test was used for inter-group comparison. Categorical data were described as case number (percentage) [n (%)], and χ^2^ test was used for inter-group comparison; when the theoretical frequency < 5, Fisher’s exact probability method was used.

Risk factor screening: ➀ Univariate analysis: Correlation analysis was performed between all potential risk factors and the occurrence of HO. Variables with *P* < 0.1 were selected for multivariate analysis to avoid missing potential independent risk factors; ➁ Multivariate analysis: A multivariate Logistic regression model was used. The dependent variable was whether HO occurred after surgery, and the independent variables were the variables selected by univariate analysis. To address potential methodological concerns regarding stepwise selection and to disentangle clinically interrelated variables, we refined our modeling strategy. While stepwise backward selection was initially performed, we also constructed a sensitivity analysis using a clinically prespecified model. This prespecified model categorized predictors into two domains: Domain 1 (Baseline/Fracture-related): age, injury mechanism, and fracture comminution; Domain 2 (Perioperative/Postoperative): soft-tissue dissection, immobilization time, and postoperative infection. Both domains were entered into the regression simultaneously to assess their independent contributions while adjusting for core confounders. Furthermore, to mitigate issues of coefficient overestimation and model overfitting associated with stepwise regression, we utilized Least Absolute Shrinkage and Selection Operator (LASSO) regression as a complementary variable selection method. Furthermore, the final model was subjected to external validation in an independent cohort, which demonstrated robust performance, suggesting that the model was not excessively overfitted to the derivation cohort. The OR and 95% confidence interval (CI) of each risk factor were calculated to identify independent risk factors.

Based on the independent risk factors screened by multivariate Logistic regression analysis, the risk prediction model was constructed by assigning values according to the regression coefficient (β) of each variable. The regression equation was generated as follows: Risk score = β_1_X_1_ + β_2_X_2_ +… + β_n_X_n_, where β is the regression coefficient and X is the assignment of each independent risk factor (dichotomous variable: yes = 1, no = 0).

Model Validation: ➀ Internal validation: Bootstrap resampling was used for internal validation with 1,000 times. For each bootstrap sample, the model was refitted, and its performance was evaluated on both the bootstrap sample itself (apparent performance) and the original cohort (test performance). The optimism-corrected AUC was then calculated as the mean difference between the apparent AUC and the test AUC across all bootstrap samples. This procedure provides a bias-adjusted estimate of model discrimination; ➁ External validation: The constructed model was applied to the external validation cohort (186 patients). The external validation AUC value was calculated to evaluate the generalization ability of the model; ➂ Performance evaluation: - Discrimination: ROC curve was drawn, and AUC value was calculated. AUC > 0.7 was considered as moderate performance, and AUC > 0.8 was considered as high performance. Meanwhile, the sensitivity, specificity, positive predictive value (PPV) and negative predictive value (NPV) corresponding to the optimal cut-off value (determined according to the maximum Youden index principle) were calculated. - Calibration: Calibration curve was drawn to intuitively present the consistency between the predicted HO incidence and the actual incidence. While the Hosmer-Lemeshow test and calibration plots were initially performed on the development cohort to assess goodness-of-fit, external calibration was subsequently evaluated using the independent validation cohort to confirm the model’s predictive accuracy in new patients. *P* > 0.05 indicated good calibration of the model. - Clinical net benefit: Decision curve analysis (DCA) was drawn to calculate the net benefit rate of the model under different risk thresholds. It was compared with the “Treat all” strategy (assuming all patients have HO and receive preventive intervention) and the “Treat none” strategy (assuming all patients do not have HO and do not receive intervention) to clarify the clinical application value of the model.

Model visualization: The rms package of R 4.2.0 software was used to convert the risk prediction model into a visual nomogram. The construction of the nomogram was based on the regression coefficients of each independent risk factor. A corresponding score scale was set for each variable. The total risk score was obtained by summing the scores of each variable. Then, the predicted probability of HO occurrence in the patient was calculated according to the “Probability of HO” scale corresponding to the total risk score, which was convenient for clinicians to quickly calculate the risk of HO occurrence in the patient.

## 3 Results

### 3.1 Baseline characteristics of the study participants

**FIGURE 1:**
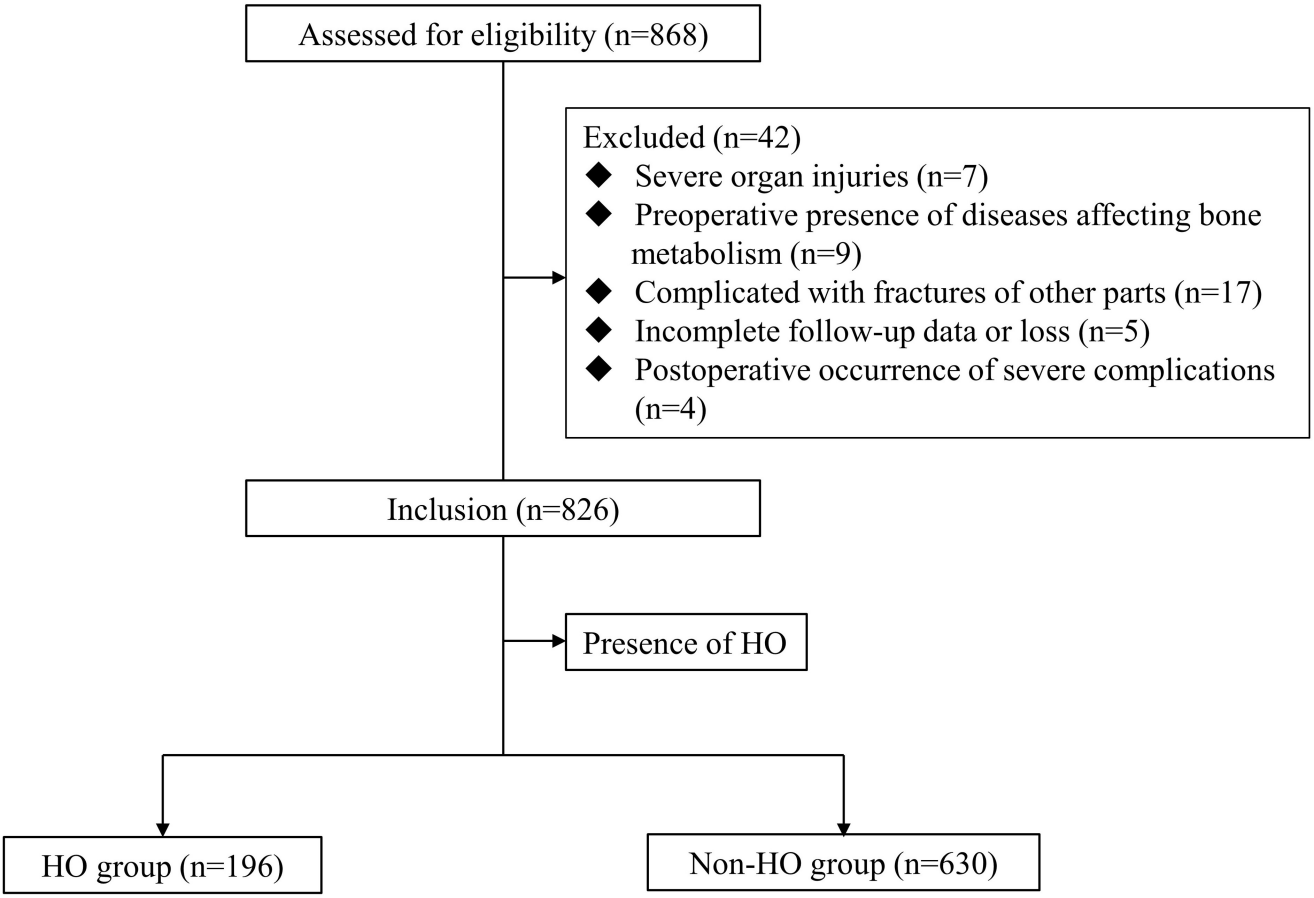
The patient flow diagram.

A total of 826 patients with distal humeral fractures who underwent ORIF were included as the main cohort. The detailed patient screening and selection process is illustrated in Figure 1. Before analysis, baseline characteristics were compared between the 826 included patients and 42 excluded patients to assess potential attrition bias. There were no significant differences in age, gender, BMI, injury mechanism, fracture type, or comorbidities between the two groups (all *P* > 0.05), indicating minimal risk of attrition bias. Among included 826 patients, 458 were male (55.4%) and 368 were female (44.6%); the age ranged from 18 to 82 years, with an average age of (52.3 ± 12.6) years; the BMI was (24.1 ± 3.5) kg/m^2^; 162 cases (19.6%) had a smoking history, and 138 cases (16.7%) had a drinking history; 78 cases (9.4%) had diabetes, and 156 cases (18.9%) had hypertension. Regarding the cause of fracture, 386 cases (46.7%) were high-energy injuries and 440 cases (53.3%) were low-energy injuries; AO/OTA classification: 218 cases (26.4%) were 13-A type, 326 cases (39.5%) were 13-B type, and 282 cases (34.1%) were 13-C type; 412 cases (49.9%) were comminuted fractures; 124 cases (15.0%) were complicated with elbow dislocation; the median time from fracture to surgery was 3 (1, 5) days. Surgery-related indicators: 528 cases (63.9%) adopted olecranon osteotomy approach, and 298 cases (36.1%) adopted lateral approach; the average operation time was (98.5 ± 26.3) minutes; the median intraoperative blood loss was 80 (50, 120) ml; 286 cases (34.6%) had extensive soft tissue dissection; the main type of internal fixation was plate and screw, with 792 cases (95.9%), and 34 cases (4.1%) were intramedullary nails; 156 cases (18.9%) underwent bone grafting; the main surgeons were chief physicians/associate chief physicians, with 586 cases (70.9%). Postoperative management indicators: 428 cases (51.8%) used NSAIDs to prevent HO; the median postoperative immobilization time was 3 (2, 4) weeks; the average start time of rehabilitation training was (14.3 ± 5.6) days; 36 cases (4.4%) had postoperative infection; 758 cases (91.8%) had drainage tubes placed, and the average indwelling time of the drainage tubes was (2.8 ± 1.1) days.

According to whether HO occurred after surgery, the patients in the main cohort were divided into the HO group (196 cases) and the non-HO group (630 cases). The results of univariate analysis of baseline data between the two groups are shown in Table 1. It can be seen from Table 1 that there were significant differences between the HO group and the non-HO group in indicators such as age, cause of fracture, fracture AO/OTA classification, whether it was a comminuted fracture, range of soft tissue dissection, postoperative immobilization time, postoperative infection and NSAIDs use (*P* < 0.05). However, there were no significant differences in gender, BMI, smoking history, drinking history, comorbidities, whether complicated with elbow dislocation, time from fracture to surgery, surgical approach, operation time, intraoperative blood loss, type of internal fixation, bone grafting, professional title of the surgeon, start time of rehabilitation training and drainage status (*P* > 0.05).

**TABLE 1:** Univariate analysis of baseline data between HO group and non-HO group.

Indicators	Total population (*n* = 826)	HO group (*n* = 196)	Non-HO group (*n* = 630)	*P*-value
Age (years), x ± s	52.3 ± 12.6	58.6 ± 11.8	50.5 ± 12.3	< 0.001
Gender, n (%)				
Male	458(55.4)	108(55.1)	350(55.6)	0.885
Female	368(44.6)	88(44.9)	280(44.4)	
BMI (kg/m^2^), x ± s	24.1 ± 3.5	24.3 ± 3.7	24.0 ± 3.4	0.356
Smoking history, n (%)				
Yes	162(19.6)	38(19.4)	124(19.7)	0.913
No	664(80.4)	158(80.6)	506(80.3)	
Drinking history, n (%)				
Yes	138(16.7)	32(16.3)	106(16.8)	0.832
No	688(83.3)	164(83.7)	524(83.2)	
Comorbidities, n (%)				
Yes	212(25.7)	52(26.5)	160(25.4)	0.686
No	614(74.3)	144(73.5)	470(74.6)	
Cause of fracture, n (%)				
High-energy injury	386(46.7)	128(65.3)	258(40.9)	< 0.001
Low-energy injury	440(53.3)	68(34.7)	372(59.1)	
AO/OTA classification, n (%)				
13-A type	218(26.4)	28(14.3)	190(30.2)	< 0.001
13-B type	326(39.5)	66(33.7)	260(41.3)	
13-C type	282(34.1)	102(52.0)	180(28.6)	
Comminuted fracture, n (%)				
Yes	412(49.9)	124(63.3)	288(45.7)	< 0.001
No	414(50.1)	72(36.7)	342(54.3)	
Complicated with elbow dislocation, n (%)				
Yes	124(15.0)	32(16.3)	92(14.6)	0.499
No	702(85.0)	164(83.7)	538(85.4)	
Time from fracture to surgery (days), M (Q1, Q3)	3(1,5)	3(1,5)	3(1,5)	0.743
Surgical approach, n (%)				
Olecranon osteotomy approach	528(63.9)	128(65.3)	400(63.5)	0.593
Lateral approach	298(36.1)	68(34.7)	230(36.5)	
Operation time (minutes), x ± s	98.5 ± 26.3	101.2 ± 27.5	97.8 ± 25.8	0.154
Intraoperative blood loss (ml), M (Q1, Q3)	80(50,120)	85(55,125)	78(48,118)	0.216
Range of soft tissue dissection, n (%)				
Extensive	286(34.6)	98(50.0)	188(29.8)	< 0.001
Limited	540(65.4)	98(50.0)	442(70.2)	
Type of internal fixation, n (%)				
Plate and screw	792(95.9)	188(95.9)	604(95.9)	1.000
Intramedullary nail	34(4.1)	8(4.1)	26(4.1)	
Bone grafting, n (%)				
Yes	156(18.9)	38(19.4)	118(18.7)	0.780
No	670(81.1)	158(80.6)	512(81.3)	
Professional title of the surgeon, n (%)				
Chief physician/Associate chief physician	586(70.9)	140(71.4)	446(70.8)	0.979
Attending physician	212(25.7)	48(24.5)	164(26.0)	
Resident physician	28(3.4)	8(4.1)	20(3.2)	
Postoperative NSAIDs use, n (%)				
Yes	428(51.8)	76(38.8)	352(55.9)	< 0.001
No	398(48.2)	120(61.2)	278(44.1)	
Postoperative immobilization time (weeks), M (Q1, Q3)	3(2,4)	4(3,5)	3(2,4)	< 0.001
Start time of rehabilitation training (days), x ± s	14.3 ± 5.6	14.8 ± 5.8	14.1 ± 5.5	0.171
Postoperative infection, n (%)				
Yes	36 (4.4)	24 (12.2)	12 (1.9)	< 0.001
No	790 (95.6)	172 (87.8)	618 (98.1)	
Postoperative drainage tube placement, n (%)				
Yes	758 (91.8)	180 (91.8)	578 (91.7)	0.964
No	68 (8.2)	16 (8.2)	52 (8.3)	
Indwelling time of drainage tube (days), x ± s	2.8 ± 1.1	2.9 ± 1.2	2.8 ± 1.1	0.324

### 3.2 Incidence of HO after ORIF of distal humeral fractures

Among the 826 patients in the main cohort, 196 cases developed HO within 6 months after surgery, with an overall incidence of 23.7%. There were significant differences in the incidence of HO among different subgroups (Table 2): The incidence of HO in patients with high-energy injuries (33.2%) was significantly higher than that in patients with low-energy injuries (15.5%); the incidence of HO in patients with 13-C type fractures (36.2%) was significantly higher than that in patients with 13-B type (20.2%) and 13-A type (12.8%); the incidence of HO in patients with comminuted fractures (30.1%) was significantly higher than that in patients with non-comminuted fractures (17.4%); the incidence of HO in patients with postoperative immobilization time ≥ 4 weeks (41.5%) was significantly higher than that in patients with immobilization time < 4 weeks (15.8%); the incidence of HO in patients with postoperative infection (66.7%) was significantly higher than that in patients without postoperative infection (21.0%).

**TABLE 2:** Comparison of postoperative HO incidence among different subgroups.

Subgroup classification	Case number (n)	HO case number (n)	Incidence (%)	*P*-value
Cause of fracture				< 0.001
High-energy injury	386	128	33.2	
Low-energy injury	440	68	15.5	
AO/OTA classification				< 0.001
13-A type	218	28	12.8	
13-B type	326	66	20.2	
13-C type	282	102	36.2	
Fracture type				< 0.001
Comminuted fracture	412	124	30.1	
Non-comminuted fracture	414	72	17.4	
Postoperative immobilization time				< 0.001
<4 weeks	598	94	15.8	
≥ 4 weeks	228	94	41.5	
Postoperative infection				< 0.001
Yes	36	24	66.7	
No	790	172	21.0	

### 3.3 Results of independent risk factors analysis for HO

Variables with *P* < 0.1 in univariate analysis (age, cause of fracture, AO/OTA classification, whether it was a comminuted fracture, range of soft tissue dissection, postoperative NSAIDs use, postoperative immobilization time, postoperative infection) were included in the multivariate Logistic regression model. Before multivariate Logistic regression analysis, collinearity test (Variance Inflation Factor, VIF) was performed on the variables included in the model. The results showed that the VIF values of all variables were between 1.02 and 1.35, which were far less than 10, indicating no significant collinearity among the variables, and they could be included in the multivariate regression model for analysis. Stepwise backward selection method was used for analysis. Finally, 6 independent risk factors for HO were identified: age ≥ 60 years, high-energy injury, comminuted fracture, extensive intraoperative soft tissue dissection, postoperative immobilization time ≥ 4 weeks and postoperative infection (Table 3). Among them, postoperative infection had the highest OR value (4.72), indicating that it had the strongest impact on the occurrence of HO; followed by high-energy injury (OR = 3.21) and postoperative immobilization time ≥ 4 weeks (OR = 3.05).

**TABLE 3:** Multivariate logistic regression analysis results of HO after ORIF of distal humeral fractures.

Variables	Assignment	Regression coefficient (β)	Standard error (SE)	OR value	95%CI	*P*-value
Age	≥ 60 years = 1, < 60 years = 0	0.958	0.186	2.86	1.98∼4.13	< 0.001
Cause of fracture	High-energy injury = 1, Low-energy injury = 0	1.166	0.203	3.21	2.25∼4.58	< 0.001
Comminuted fracture	Yes = 1, No = 0	0.928	0.195	2.53	1.76∼3.64	< 0.001
Range of soft tissue dissection	Extensive = 1, Limited = 0	0.779	0.198	2.18	1.52∼3.13	< 0.001
Postoperative immobilization time	≥ 4 weeks = 1, < 4 weeks = 0	1.115	0.212	3.05	2.11∼4.40	< 0.001
Postoperative infection	Yes = 1, No = 0	1.552	0.318	4.72	2.68∼8.32	< 0.001
AO/OTA classification	13-B type = 1, 13-A type = 0; 13-C type = 1, 13-A type = 0	0.325 (13-B type); 0.512 (13-C type)	0.241 (13-B type); 0.268 (13-C type)	1.384 (13-B type); 1.668 (13-C type)	0.876∼2.182 (13-B type); 0.985∼2.821 (13-C type)	0.178 (13-B type); 0.057 (13-C type)
Postoperative NSAIDs use	Yes = 1, No = 0	-0.428	0.215	0.652	0.423∼1.005	0.053
Constant term	–	-2.364	0.286	0.093	–	< 0.001

### 3.4 Construction and visualization of the risk prediction model

Based on the results of multivariate Logistic regression analysis, a risk prediction model was constructed. The regression equation was: Risk score = 0.958 × age ( ≥ 60 years = 1, < 60 years = 0) + 1.166 × injury type (high-energy = 1, low-energy = 0) + 0.928 × fracture type (comminuted = 1, non-comminuted = 0) + 0.779 × range of soft tissue dissection (extensive = 1, limited = 0) + 1.115 × postoperative immobilization time ( ≥ 4 weeks = 1, < 4 weeks = 0) + 1.552 × postoperative infection (yes = 1, no = 0).

**FIGURE 2:**
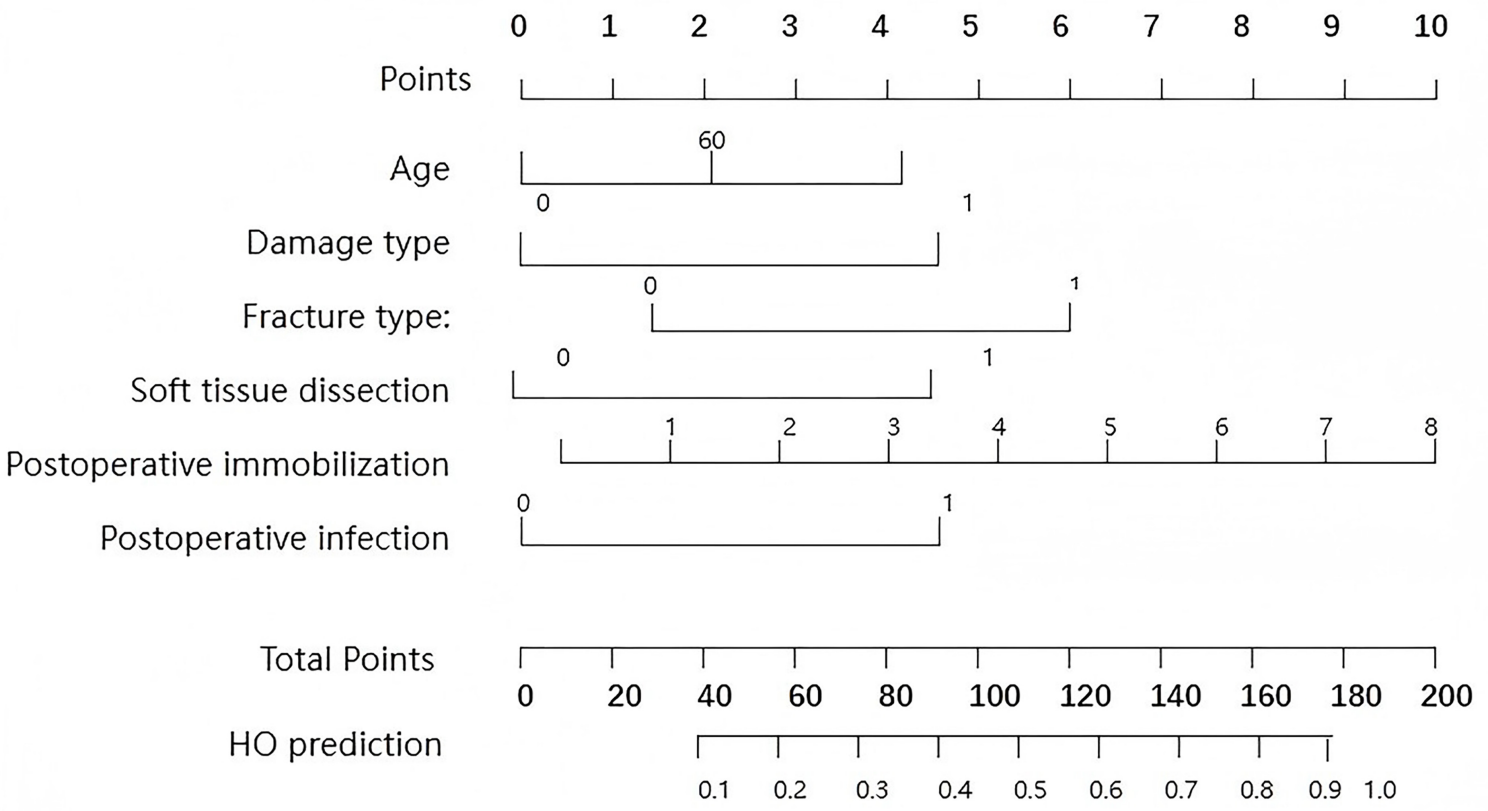
Nomogram for predicting heterotopic ossification (HO) after open reduction and internal fixation (ORIF) of distal humeral fractures.

The rms package of R 4.2.0 software was used to visualize the model and construct a nomogram (Figure 2). The nomogram included 6 independent risk factors. Each variable corresponded to a scale line, and the values on the scale line were the scores of the variable. When using, first determine the score corresponding to the patient’s actual situation on each variable scale line, sum the scores of all variables to get the total risk score, and then read the predicted probability of HO occurrence in the patient after surgery through the “Probability of HO” scale corresponding to the total risk score. For example, a 65-year-old patient (age score 2.8 points) with high-energy injury (score 3.4 points), comminuted fracture (score 2.7 points), extensive intraoperative soft tissue dissection (score 2.3 points), postoperative immobilization for 4 weeks (score 3.3 points) and no postoperative infection (score 0 points) had a total risk score of 2.8 + 3.4 + 2.7 + 2.3 + 3.3 + 0 = 14.5 points, and the corresponding predicted probability of HO occurrence was about 75%.

### 3.5 Validation results of the risk prediction model

#### 3.5.1 Discrimination validation

**FIGURE 3:**
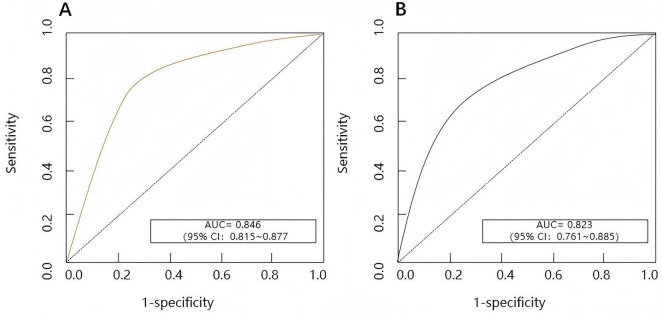
Receiver operating characteristic (ROC) curves of the predictive model. **(A)** The internal validation AUC of the model was 0.846. **(B)** The external validation AUC was 0.823.

The apparent AUC of the model fitted on the entire development cohort was 0.882. After correction for optimism using Bootstrap resampling (1,000 iterations), the optimism-corrected internal validation AUC was 0.846 (95% CI: 0.815∼0.877) (Figure 3A), indicating good discrimination. According to the maximum Youden index principle (Youden index = 0.582), the optimal cut-off value of the model was determined to be 0.32. At this time, the sensitivity of the model was 78.6%, the specificity was 79.6%, the positive predictive value was 52.3%, and the negative predictive value was 92.1%.

In the external validation cohort (*n* = 186), the overall incidence of HO was 22.6% (42/186). The baseline characteristics of this cohort were largely comparable to the main derivation cohort. Patients had a mean age of 51.8 ± 13.1 years, with 55.9% (104/186) being male. The distribution of key risk factors was as follows: high-energy injury 45.2% (84/186), comminuted fracture 48.4% (90/186), and postoperative infection 4.8% (9/186). The AUC value of the ROC curve of the model was 0.823 (95% CI: 0.761∼0.885) (Figure 3B), the sensitivity was 76.3%, the specificity was 78.9%, the positive predictive value was 49.2%, and the negative predictive value was 91.5%, indicating that the model had good generalization ability.

#### 3.5.2 Calibration validation

**FIGURE 4:**
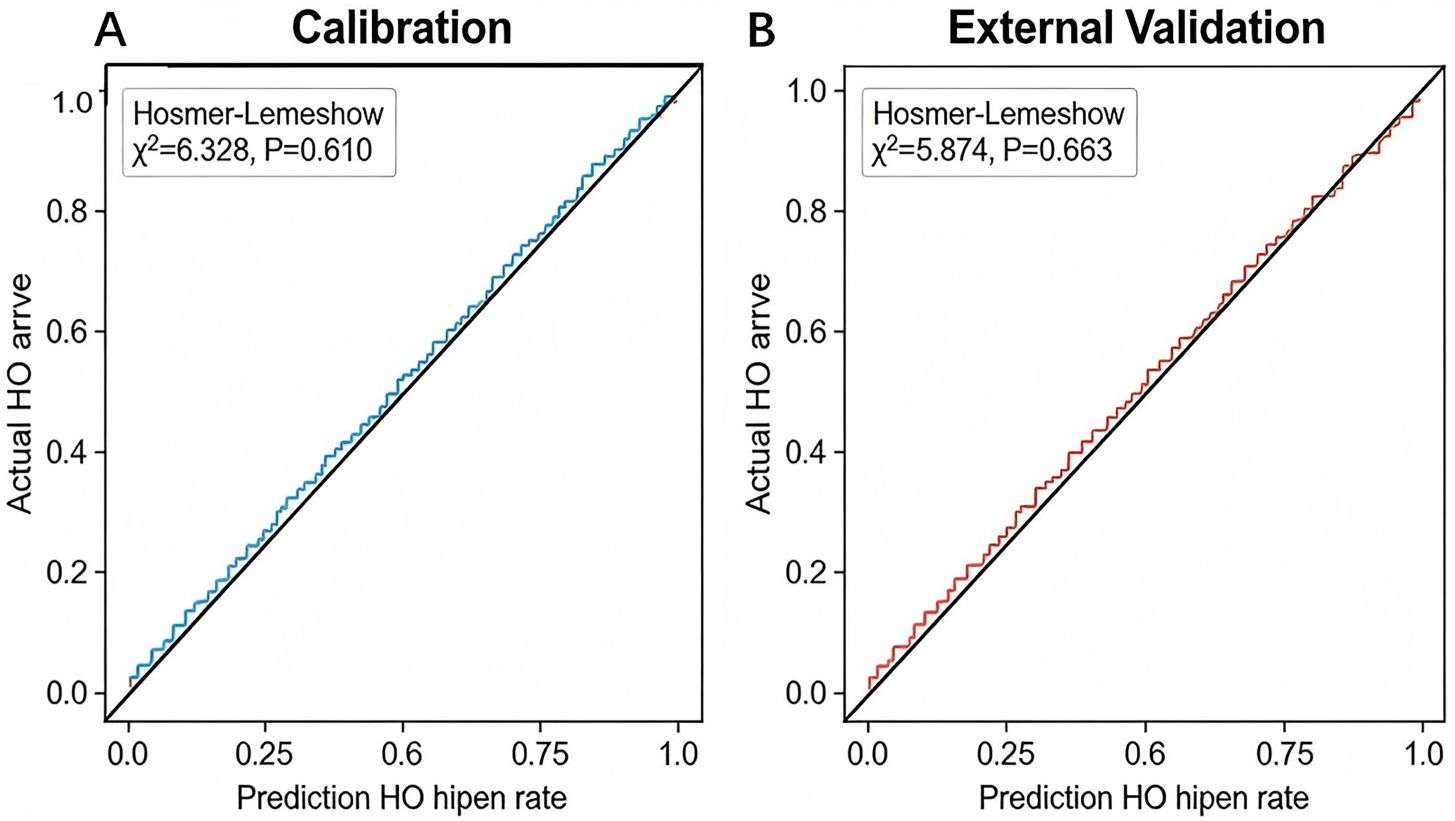
Calibration curves of the predictive model. **(A)** The calibration curve of the main cohort showed that the Hosmer-Lemeshow test result was χ^2^ = 6.328, *P* = 0.610, indicating that the model had good calibration. **(B)** The calibration curve of the external validation cohort showed that the Hosmer-Lemeshow test result was χ^2^ = 5.874, *P* = 0.663, which further confirmed the calibration reliability of the model.

The calibration curve of the main cohort showed that the predicted HO incidence of the model was in good agreement with the actual incidence (Figure 4A). The Hosmer-Lemeshow test result was χ^2^ = 6.328, *P* = 0.610, indicating that the model had good calibration, and the predicted probability was highly consistent with the actual risk.

The calibration curve of the external validation cohort also showed that the predicted incidence was in good agreement with the actual incidence (Figure 4B). The Hosmer-Lemeshow test result was χ^2^ = 5.874, *P* = 0.663, which further confirmed the calibration reliability of the model.

#### 3.5.3 Clinical net benefit validation

**FIGURE 5:**
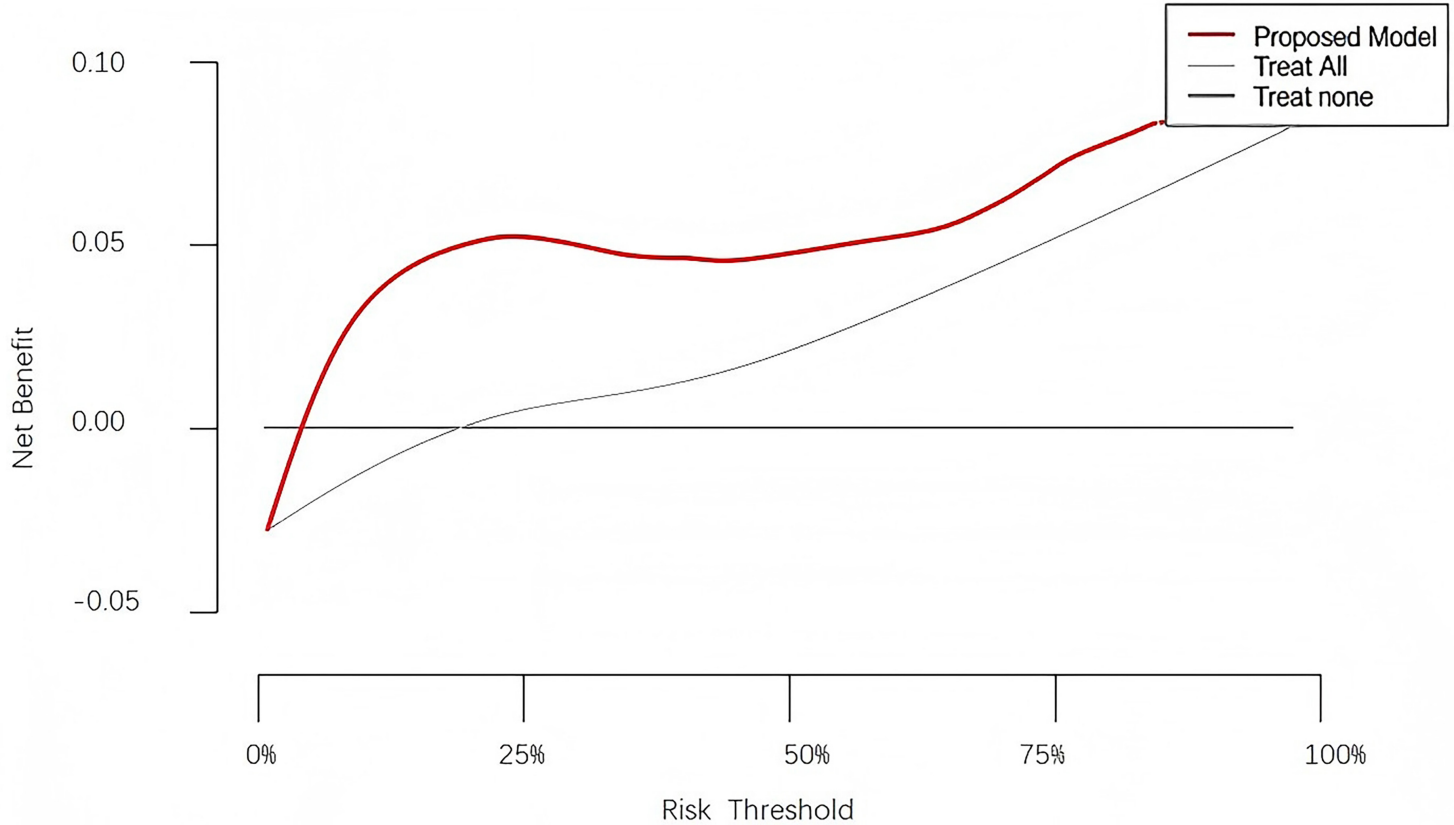
Decision curve analysis (DCA) of the predictive model.

The decision curve analysis (DCA) was performed to evaluate the clinical utility of the model (Figure 5). The results demonstrated that within a threshold probability range of 8–75%, the net benefit of using the prediction model was consistently higher than that of the “Treat all” and “Treat none” strategies. While these findings suggest the model provides a favorable trade-off between true positives and false positives, the clinical utility should be interpreted within the context of this specific threshold range. It is necessary to avoid over-interpreting these results and regarding them as an absolute proof of clinical effectiveness, as this model is primarily used as an exploratory tool for risk stratification rather than as a substitute for individualized clinical judgment.

### 3.6 Sensitivity analysis

To mitigate potential bias from confounding by indication, we excluded postoperative NSAID use from the primary model and performed a stratified analysis. As shown in Table 4, in the subgroup of patients who did not receive NSAIDs (*n* = 398), the independent risk factors remained consistent with the primary model, with high-energy injury (OR = 3.45, 95% CI: 2.28–5.22) and postoperative infection (OR = 5.12, 95% CI: 2.85–9.21) remaining significant predictors. The incidence of HO was significantly lower in the NSAID-user subgroup (17.8% vs. 30.2%, *P* < 0.001), confirming its protective association without compromising the integrity of the baseline risk model.

**TABLE 4:** Sensitivity analysis results of risk factors after excluding NSAID usage and domain grouping analysis.

Analysis grouping	Influencing factors	β	OR	95% CI	*P*-value
Subgroup without NSAIDs (*n* = 398)	Age ≥ 60y	0.972	2.91	2.01∼4.21	<0.001
	High-energy injury	1.238	3.45	2.28∼5.22	<0.001
	Comminuted fracture	0.941	2.56	1.74∼3.76	<0.001
	Extensive soft tissue dissection	0.793	2.21	1.50∼3.25	<0.001
	Postoperative immobilization ≥ 4w	1.132	3.10	2.14∼4.49	<0.001
	Postoperative infection	1.633	5.12	2.85∼9.21	<0.001
Domain 2 adjusted for Domain1 (Baseline/fracture factors)	Age ≥ 60y	0.949	2.59	1.81∼3.71	<0.001
	High-energy injury	1.183	3.27	2.31∼4.63	<0.001
	Comminuted fracture	0.908	2.48	1.72∼3.58	<0.001
Domain1 adjusted for Domain2 (Perioperative/postoperative factors)	Extensive soft tissue dissection	0.798	2.22	1.55∼3.18	<0.001
	Postoperative immobilization ≥ 4w	1.102	3.01	2.09∼4.34	<0.001
	Postoperative infection	1.515	4.55	2.58∼8.02	<0.001
LASSO regression screening results	Age ≥ 60y	0.951	2.83	1.96∼4.09	<0.001
	High-energy injury	1.159	3.19	2.23∼4.55	<0.001
	Comminuted fracture	0.922	2.51	1.74∼3.61	<0.001
	Extensive soft tissue dissection	0.773	2.17	1.51∼3.11	<0.001
	Postoperative immobilization ≥ 4w	1.109	3.03	2.09∼4.37	<0.001
	Postoperative infection	1.547	4.69	2.66∼8.26	<0.001

Smoking history was compressed to zero coefficient in LASSO regression and not included in final predictors; HO incidence: NSAID unused group 30.2%, NSAID used group 17.8%, *P* < 0.001.

To disentangle predictive signals from variables lying on the same causal pathway, we constructed a clinically prespecified model categorizing predictors into two domains: Domain 1 (Baseline/Fracture-related): Age, high-energy injury, and comminuted fracture; Domain 2 (Perioperative/Postoperative): Extensive soft-tissue dissection, postoperative immobilization time ≥ 4 weeks, and postoperative infection. After adjusting for Domain 2 variables, the association between fracture comminution (OR = 2.48, 95% CI: 1.72–3.58) remained significant. Similarly, after adjusting for Domain 1 variables, extensive soft-tissue dissection (OR = 2.22, 95% CI: 1.55–3.18) and postoperative infection (OR = 4.55, 95% CI: 2.58–8.02) remained strong predictors. This suggests that while these variables are clinically sequential, their predictive values can be distinguished.

To address the instability of stepwise regression and prevent overfitting, we implemented LASSO regression with 10-fold cross-validation. The LASSO model selected the same set of predictors as the primary stepwise model, with the exception of smoking history, which was shrunk to zero. The consistency between the stepwise and LASSO models validates the stability of the identified risk factors.

## 4 Discussion

Based on multi-center retrospective cohort data, this study systematically analyzed the risk factors of HO after ORIF of distal humeral fractures and constructed a visual risk prediction model. The results showed that the overall incidence of HO was 23.7%. Age ≥ 60 years, high-energy injury, comminuted fracture, extensive intraoperative soft tissue dissection, postoperative immobilization time ≥ 4 weeks and postoperative infection were independent risk factors for HO. The constructed model had good discrimination, calibration and clinical net benefit, providing a preliminary visualization tool for HO risk stratification.

### 4.1 Interpretation of core research results

#### 4.1.1 Analysis of HO incidence

The overall incidence of HO in this study was 23.7%, which was consistent with the results of most previous studies (15∼40%)(6–8). This further confirms that HO is a common complication after ORIF of distal humeral fractures. Subgroup analysis showed that the incidence of HO was significantly higher in patients with high-energy injury, 13-C type fracture, comminuted fracture, postoperative immobilization time ≥ 4 weeks and postoperative infection. This is closely related to the pathogenesis of HO. High-energy injuries and comminuted fractures are often accompanied by more severe soft tissue damage and local inflammatory response, and inflammatory response is a key initiating factor for HO (11); 13-C type fracture is a complex intra-articular fracture, which is difficult to reduce surgically, and the range of soft tissue dissection may be wider, further aggravating local tissue damage; long-term postoperative immobilization will lead to poor blood circulation around the joint, accumulation of inflammatory factors, and reduce the mechanical inhibition of osteoblasts, promoting the formation of heterotopic bone (24); the chronic inflammatory microenvironment caused by postoperative infection will continuously activate osteoblast differentiation, significantly increasing the risk of HO (25).

#### 4.1.2 Clinical significance and mechanism discussion of independent risk factors

➀ Age ≥ 60 years: This study found that age ≥ 60 years was an independent risk factor for HO (OR = 2.86), which was consistent with the results of some previous studies (14, 15). Elderly people have disorders of bone metabolism, imbalance between osteoblast and osteoclast functions. At the same time, elderly people often have underlying diseases, poor local blood circulation, and longer duration of inflammatory response after fracture. These factors together increase the risk of HO (26). In addition, the compliance of elderly patients with postoperative rehabilitation training may be low, and insufficient joint activity may also indirectly promote the formation of HO.

➁ High-energy injury: High-energy injury is an important risk factor for HO (OR = 3.21). High-energy injury not only leads to more severe fractures (such as comminuted fractures) but also causes severe contusion of soft tissues around the joint and rupture of blood vessels, resulting in hematoma formation. During hematoma organization, a large number of inflammatory factors (such as tumor necrosis factor-α, interleukin-6) and bone morphogenetic proteins are released. These factors will recruit mesenchymal stem cells and induce their differentiation into osteoblasts, thereby forming heterotopic bone tissue (27).

➂ The incidence of HO in patients with comminuted fractures was significantly increased (OR = 2.53), which is closely related to the pathological characteristics of comminuted fractures. Comminuted fractures have many fracture fragments, which are difficult to reduce. The operation time is longer, the range of soft tissue dissection is wider, and the local tissue damage is more severe; at the same time, the stability of the fracture end is poor, and the postoperative immobilization time may be longer. These factors all increase the risk of HO (28). In addition, the callus formation process after comminuted fractures is more active, and excessive callus growth may also invade the surrounding soft tissues, forming HO.

➃ Extensive intraoperative soft tissue dissection: Extensive intraoperative soft tissue dissection is an independent risk factor for HO (OR = 2.18). The soft tissues around the elbow joint are rich in mesenchymal stem cells. Excessive dissection will expose these stem cells to inflammatory factors and bone morphogenetic proteins released by the fracture end, inducing their differentiation into osteoblasts; at the same time, extensive dissection will damage the blood circulation around the joint, leading to local tissue hypoxia and aggravated inflammatory response, further promoting the formation of HO (29). This result suggests that unnecessary soft tissue dissection should be minimized during surgery, and the ligaments and soft tissues around the joint should be protected to reduce the risk of HO.

➄ Postoperative immobilization time ≥ 4 weeks: Postoperative immobilization time ≥ 4 weeks significantly increased the risk of HO (OR = 3.05), which was consistent with previous research conclusions (30). Joint activity can regulate local blood circulation through mechanical stimulation, promote the clearance of inflammatory factors, and inhibit the excessive differentiation of osteoblasts. Long-term postoperative immobilization will lead to adhesion of tissues around the joint, accumulation of inflammatory factors, increased differentiation of mesenchymal stem cells into osteoblasts, and thus promote the formation of heterotopic bone. Therefore, early standardized rehabilitation training is crucial on the premise of stable fracture end.

➅ Postoperative infection: Postoperative infection is the strongest risk factor for HO (OR = 4.72), which is consistent with related studies. Postoperative infection will trigger a persistent local inflammatory response. A large number of inflammatory factors in the inflammatory microenvironment will activate the proliferation and differentiation of osteoblasts, and inhibit the function of osteoclasts, leading to abnormal bone tissue proliferation (31, 32). In addition, infection will damage the local tissue repair process, leading to abnormal hematoma organization and promoting the formation of HO. Therefore, preventing postoperative infection is one of the key measures to reduce the risk of HO.

It is worth noting that this study did not find an association between gender, BMI, smoking history, drinking history, comorbidities, surgical approach, operation time, bone grafting and other factors and the occurrence of HO. This may be related to the large sample size and the control of confounding factors in multivariate analysis. For example, some previous studies believed that smoking may increase the risk of HO (33), but there was no significant difference in smoking history between the two groups in this study. This may be related to the low smoking rate of the included patients (19.6%) or the influence of smoking on HO being masked by other stronger risk factors, such as, postoperative infection, high-energy injury. In addition, it is crucial to interpret the “independent risk factors” identified by multivariable logistic regression with caution. While statistical independence was achieved (all VIF values < 1.35), several variables are inherently clinically interrelated and may lie on the same causal pathway. For instance, more complex fractures (e.g., AO/OTA 13-C type) often necessitate wider surgical exposure, leading to more extensive soft-tissue dissection and potentially longer postoperative immobilization. Low VIF values indicate a lack of multicollinearity but do not fully exclude such clinically meaningful dependencies. Therefore, the factors identified herein should be viewed as associations and predictors rather than definitive causal agents.

#### 4.1.3 Evaluation of the risk prediction model’s performance

The risk prediction model constructed in this study included 6 independent risk factors. The internal validation AUC was 0.846, and the external validation AUC was 0.823, both greater than 0.8, indicating that the model had good discrimination; the calibration curve showed that the predicted probability was in good agreement with the actual incidence (Hosmer-Lemeshow test *P* > 0.05), indicating that the model had reliable calibration; the DCA results showed that within the risk threshold range of 8–75%, the clinical net benefit of the model was superior to the “Treat all” and “Treat none” strategies, confirming that the model had high clinical application value. Compared with existing related models (20, 21), the model in this study had the following advantages: ➀ Including multi-center large sample data to improve the representativeness and generalization ability of the model; ➁ Comprehensively covering multi-dimensional risk factors with strong pertinence; ➂ Adopting strict internal and external validation methods with comprehensive performance evaluation; ➃ Constructing a visual nomogram with simple operation, convenient for clinicians to use quickly.

### 4.2 Comparison with domestic and foreign related studies

The risk factors such as high-energy injury, comminuted fracture, long postoperative immobilization time and postoperative infection screened in this study were consistent with the results of most previous studies (18, 24, 25, 30), further confirming the important role of these factors in the occurrence of HO. However, regarding the impact of age, some studies have not found an association between age and HO (16, 17), which may be related to the age distribution of the study population, sample size and control of confounding factors. For example, some studies included a higher proportion of young patients or did not control confounding factors such as postoperative immobilization time and infection, leading to the impact of age being masked. In addition, this study did not find a correlation between surgical timing and HO, which was inconsistent with the conclusion of some studies (18) that early surgery can reduce the risk of HO. This may be attributed to the short median time from fracture to surgery in this study (3 days), most patients received early surgery, and the difference between groups was small, so the impact of surgical timing was not shown.

At present, there are very few specific risk prediction models for HO after ORIF of distal humeral fractures. Most of the existing models are general models for HO after various fractures. For example, the risk prediction model for HO after fracture constructed by Wahl et al. (20) included variables such as fracture type and surgical approach, but the sample size was small and no external validation was performed; the model for HO after elbow joint surrounding fracture constructed by Lauder and Richard (21) included fewer variables, only fracture type and soft tissue injury degree, and the model performance was limited. In contrast, the model in this study is a specific model for HO after ORIF of distal humeral fractures. It includes 6 targeted independent risk factors, has a larger sample size, adopts multi-center internal and external validation, has higher model performance (AUC > 0.8), and constructs a visual nomogram with stronger clinical applicability.

Notably, while the constructed nomogram provides a convenient visual tool to quantify individual patient risk, clinicians should interpret its predictions with caution. The model reflects associations observed in our specific cohort and may not fully account for variations in surgical technique or postoperative care pathways across different institutions. Therefore, the current nomogram should be considered a hypothesis-generating tool to identify high-risk patients for closer monitoring, rather than a replacement for comprehensive clinical judgment.

### 4.3 Limitations of the study

This study had certain limitations: ➀ The study design was a retrospective cohort study. Although multi-center data were used and some confounding factors were controlled, there may still be selection bias and information bias such as, incomplete recording of some medical records; ➁ Potential risk factors such as gene polymorphism and serum inflammatory factor levels were not included, which may affect the model’s performance. Regarding model validation, while the Hosmer-Lemeshow test indicated good calibration in the development cohort, this assessment was performed on the same dataset used for model building. Although external calibration was evaluated in an independent cohort, we acknowledge that a more rigorous internal calibration validation using bootstrap methods would have further strengthened the findings; ➂ The follow-up time was 6 months, and the long-term evolution of HO was not observed. Some patients may develop HO after 6 months of surgery; ➃ The validation cohort only came from a single hospital and was used for temporal external validation rather than independent geographic external validation. Its relatively small sample size may affect the assessment of the model’s generalizability; ➄ Regarding the outcome measurement, HO was treated as a binary outcome without grading its severity or anatomical location using established classification systems such as the Hastings and Graham classification. While this approach is suitable for developing a risk prediction model focused on incidence, it limits our ability to correlate the predicted risk with the actual clinical burden of HO (e.g., specific motion deficits). Consequently, the clinical interpretability of the model regarding “how bad” the HO will be is limited.

### 4.4 Future research directions

Future studies can be carried out as follows: ➀ Conduct prospective multi-center studies to expand the sample size, include more potential risk factors such as gene polymorphism and serum inflammatory factors, further verify and optimize the existing model; ➁ Further explore the molecular mechanism of each independent risk factor leading to HO (inflammatory pathway, osteogenic differentiation regulatory pathway), so as to provide a theoretical basis for the development of new prevention targets; ➂ Based on the identification results of high-risk groups in this study, carry out targeted preventive intervention clinical trials to verify the effectiveness and safety of intervention measures such as NSAIDs and rehabilitation training programs; ➃ Combine artificial intelligence algorithms (machine learning, deep learning) to construct a more efficient risk prediction model, and combine the model with clinical information systems to develop convenient clinical decision support tools, so as to improve the clinical application efficiency of the model.

## 5 Conclusion

Age ≥ 60 years, high-energy injury, comminuted fracture, extensive intraoperative soft tissue dissection, postoperative immobilization time ≥ 4 weeks and postoperative infection are the core independent risk factors for HO after ORIF of distal humeral fractures. The risk prediction model (including the nomogram) constructed in this study demonstrated promising discriminative ability and calibration in the derivation cohort, with acceptable generalizability in a temporal validation cohort. While the model provides a preliminary and accessible tool for clinicians to stratify patients at risk of HO after ORIF of distal humeral fractures, caution should be exercised when applying it to broader populations. Further large-scale, multi-center studies with geographically distinct external validation are required to confirm its clinical utility before it can be considered a definitive risk assessment tool.

## Data Availability

The original contributions presented in this study are included in the article/supplementary material, further inquiries can be directed to the corresponding author.
